# Purinergic and Adenosinergic Signaling in Pancreatobiliary Diseases

**DOI:** 10.3389/fphys.2022.849258

**Published:** 2022-03-14

**Authors:** Erika Y. Faraoni, Cynthia Ju, Simon C. Robson, Holger K. Eltzschig, Jennifer M. Bailey-Lundberg

**Affiliations:** ^1^Department of Anesthesiology, Center for Perioperative Medicine, McGovern Medical School, The University of Texas Health Science Center at Houston, Houston, TX, United States; ^2^Departments of Internal Medicine and Anesthesiology, Center for Inflammation Research, Beth Israel Deaconess Medical Center and Harvard Medical School, Boston, MA, United States

**Keywords:** biliary cancers, pancreatic cancer, immune suppression, hypoxia, CD73, CD39

## Abstract

Adenosine 5'-triphosphate (ATP), other nucleotides, and the nucleoside analogue, adenosine, all have the capacity to modulate cellular signaling pathways. The cellular processes linked to extracellular purinergic signaling are crucial in the initiation, evolution, and resolution of inflammation. Injured or dying cells in the pancreatobiliary tract secrete or release ATP, which results in sustained purinergic signaling mediated through ATP type-2 purinergic receptors (P2R). This process can result in chronic inflammation, fibrosis, and tumor development. In contrast, signaling *via* the extracellular nucleoside derivative adenosine *via* type-1 purinergic receptors (P1R) is largely anti-inflammatory, promoting healing. Failure to resolve inflammation, as in the context of primary sclerosing cholangitis or chronic pancreatitis, is a risk factor for parenchymal and end-organ scarring with the associated risk of pancreatobiliary malignancies. Emerging immunotherapeutic strategies suggest that targeting purinergic and adenosinergic signaling can impact the growth and invasive properties of cancer cells, potentiate anti-tumor immunity, and also block angiogenesis. In this review, we dissect out implications of disordered purinergic responses in scar formation, end-organ injury, and in tumor development. We conclude by addressing promising opportunities for modulation of purinergic/adenosinergic signaling in the prevention and treatment of pancreatobiliary diseases, inclusive of cancer.

## Introduction

Relationships between inflammation, wound-healing, scarring, and cancer were first proposed in the middle of the 19th century by Virchow (Dvorak, 1986). Currently, inflammation is recognized as a canonical hallmark of cancer given roles linked to the various stages of tumor development and in impacting responses to therapy (Greten and Grivennikov, 2019). Inflammation appears tightly regulated by the release of inflammatory mediators inclusive of extracellular purines, heterocyclic aromatic organic compounds, and fundamental biochemical constituents of purinergic signaling (Miller and Urey, 1959). ATP is the vital purine nucleotide generated by glycolysis and oxidative phosphorylation and provides the intracellular energy currency fundamental for biological processes. Typical intracellular stores of ATP are in the range of 5–8 mM; however, several stimuli trigger heightened levels of ATP release by various cell types. This process increases local extracellular ATP concentrations to tens or even hundreds of micromoles per liter in inflamed or hypoxic tissues, such as in the tumor microenvironment (Di Virgilio et al., 2018a). Elevated extracellular ATP levels during pathophysiological conditions, such as tissue stress, necrosis, hypoxia, platelet activation, and vascular thrombosis, directly modulate recruitment and function of innate immune cells by exerting signals through purinergic receptors (Di Virgilio et al., 2018a). In this review, we consider the implications of dysregulated purinergic signaling in the evolution of pancreatobiliary diseases and in cancer.

## Deleterious vs. Beneficial Elements of Purinergic and Adenosine Signaling

**Figure 1:**
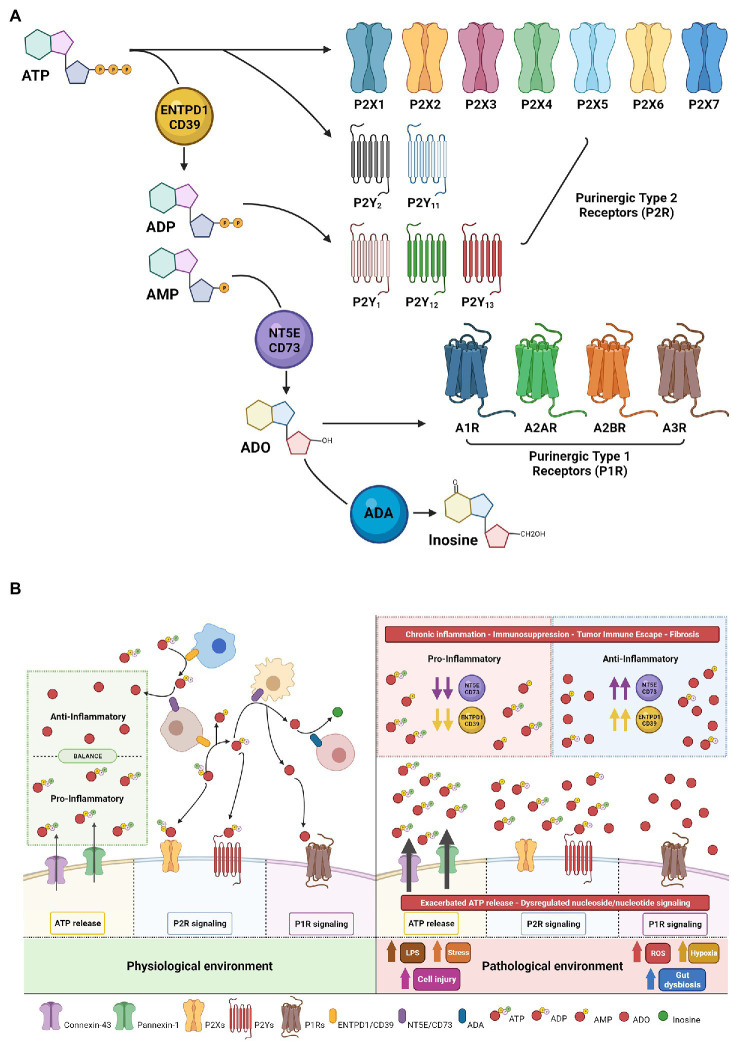
Purinergic signaling mechanisms—receptors and ecto-enzymes. **(A)** Extracellular nucleotides, nucleosides and specific receptors. Extracellular ATP initiates vascular and immune cellular signaling through purinergic-type 2 receptors (classified as P2X ligand-gated channels and P2Y—G protein-coupled receptors). Enzymatic conversion of extracellular ATP to ADP/AMP by ENTPD1/CD39 modulates nucleotide/nucleoside signaling *via* ADP generation with binding and activation of selective P2Ys receptors and/or followed by AMP conversion to adenosine (ADO) by NT5E/CD73. ADO initiates adenosinergic/nucleoside-mediated signaling through purinergic-type 1 receptors P1R or can be degraded by ADA to inosine, resulting in termination of adenosine signaling. **(B)** Beneficial vs. Deleterious Purinergic Signaling Responses in Physiological and Pathological States. With physiological quiescent conditions, low-level nanomolar concentrations of extracellular ATP in the microenvironment are essential to fine-tune and preserve cell functionality. Tightly controlled ATP conversion and purinergic signaling enable co-ordination of signals in the extracellular compartment allowing tissue-specific homeostatic functions (Left). In the setting of inflammation and other pathological states, injured cells increase ATP release, which then achieve millimolar concentrations in the extracellular compartment, promoting cellular activation and strong pro-inflammatory responses. Sustained inflammation alters the nature of purinergic signaling through P2 and P1 receptors and may promote chronic injury. In this context, ADO generation can at first exhibit beneficial cytoprotective and anti-inflammatory functions through P1 receptors to help mitigate ATP exacerbated signaling; however, in the presence of high CD39/CD73 expression and high substrate levels of extracellular nucleotides, consequent ADO signaling triggers tumor immune escape, angiogenesis, excessive fibrosis and detrimental immunosuppression (Right).

Decades of research have indicated extracellular ATP is pro-inflammatory, whereas the primary metabolite, adenosine, is largely anti-inflammatory. Integrated ATP-directed purinergic and adenosine-mediated signaling pathways appear imperative for appropriate responses to injury, healing, and subsequent tissue repair (Figure 1A).

Seminal work by Dr. Burnstock described novel ATP receptors, which were denoted Purinergic-type 2 receptors (P2R; Burnstock, 2006). In the extracellular environment, ATP-mediated purinergic signaling is initiated when ATP binds and activates P2R resulting in context and cell type-dependent responses. To date, eight P2YR (P2Y_1/2/4/6/11/12/13/14_) and seven P2XR (P2X1-7) members have been identified in this family (Figure 1B).

P2YRs are metabotropic receptors that act as G protein-coupled receptors (GPCR), while P2XRs are nucleotide-gated ion channels and are more commonly known as ionotropic receptors (Idzko et al., 2014). Unlike P2YR that can bind several nucleoside tri- and di-phosphates, P2XR are ATP-selective. P2XR affinity is generally in the low micromolar range and highly sensitive to sudden changes in local ATP concentrations. P2X7R is the exception and has a remarkably high tetrabasic ATP activation threshold in the millimolar range (North and Surprenant, 2000; Magistroni et al., 2019). Active release of ATP to the extracellular compartment is tightly mediated by connexins, pannexins, and exocytosis, which are predominantly regulated by P2XR-dependent feedback loops (Di Virgilio et al., 2020; Li et al., 2020). Heightened ATP stimulation of P2X7 triggers opening of a non-selective plasma membrane pore, known as a macropore, which promotes the release of large hydrophilic molecules (Di Virgilio et al., 2018b). Macropore activation and conductance occur as a pathophysiological function of P2X7 (Pippel et al., 2017) and can result in membrane depolarization and cell death (Harkat et al., 2017). Isoforms of P2X7R have been described that impact macropore formation including nfP2X7, an isoform with conformational changes resulting in loss of the capacity for macropore formation (Pegoraro et al., 2021). This isoform is localized to the cytosol but can be translocated to the plasma membrane after cellular exposure to high ATP concentrations (Barden et al., 2003). Strategies to rescue macropore formation are promising therapeutic avenues as these adaptations are proposed to occur in tumor microenvironments (Lara et al., 2020).

Adenosine, once available in the extracellular space, signals through the Purinergic-type 1 receptor (P1R) family, which consists of four GPCR: ADORA (A_1_R, A_2A_R, A_2B_R, and A_3_R; Figure 1A; Borea et al., 2018). P1 receptor activation on epithelial or immune cells modulates intracellular cAMP levels and contributes to anti-inflammatory and immunosuppressive responses, dampening ATP-triggered responses by P2 receptors and contributing to resolution of injury (Figure 1B; Colgan, 2015).

Under homeostatic conditions, low levels of extracellular ATP are rapidly converted through the catalytic actions of ectonucleotidases. Tandem-linked enzymatic activities of ectonucleoside triphosphate diphosphohydrolase 1 (ENTPD1/CD39) decrease extracellular ATP and ADP levels through conversion to AMP, followed by ecto-5′-nucleotidase (CD73) catalyzed adenosine generation. Under hypoxic conditions, adenosine signaling elicits tissue-protective effects and coordinates reparative mechanisms through inhibition of leukocyte cell recruitment and reduced production of pro-inflammatory cytokines. Adenosine levels are in turn regulated by cellular uptake by equilibrative nucleoside transporters (ENTs; Eltzschig et al., 2005; Rose et al., 2011; Wang et al., 2021a). In humans, nucleoside transporters are encoded by *SLC28* and *SLC29*. The *SLC28* family includes three human Concentrative Nucleoside Transporters (hCNT1, hCNT2, and hCNT3) which have characteristic transporter properties, whereas the *SLC29* family comprises four human Equilibrative Nucleoside Transporters (hENT1, hENT2, hENT3, and hENT4) which are regulators of nucleoside pools and purinergic signaling (Pastor-Anglada and Pérez-Torras, 2018). In addition to cellular uptake, adenosine levels are regulated by the catalytic activity of adenosine deaminase (ADA) that terminates extracellular and intracellular adenosine signaling by irreversibly degrading adenosine to inosine (Wiginton et al., 1981; Idzko et al., 2014). However, when these processes are overwhelmed, sustained adenosine-mediated signaling exacerbates immunosuppressive states, resulting in immune exhaustion. Tumors arising in the pancreatobiliary tract have elevated CD39 and CD73, placing CD39 or CD73 inhibitors as high priority candidates for reversing immune suppression in these lethal malignancies (Sciarra et al., 2019).

## Hypoxia-Driven Adenosinergic Signaling

Pancreatobiliary malignancies including cholangiocarcinoma and pancreatic ductal adenocarcinoma have profoundly hypoxic and immunosuppressive tumor microenvironments. Hypoxia has been shown to enhance the invasive and malignant properties of these malignant cells (Lee et al., 2016; Yu et al., 2020). Their hypoxic microenvironments are important in the context of nucleoside signaling as hypoxia-mediated adenosine signaling is central for immunosuppression and immune escape (Bastid et al., 2015; Hayes et al., 2015). Under hypoxic conditions, P1-mediated adenosine signaling is boosted by the concerted and upregulated ecto-enzymatic activity of CD39 and CD73 on epithelial cells. CD73 transcriptional activity is regulated by a Hypoxia Response Element (HRE) located in the promoter region of *NT5E* (CD73). This HRE element allows HIF-1α to directly regulate CD73 in epithelial cells under hypoxic conditions (Synnestvedt et al., 2002). In addition, under hypoxic conditions in epithelial cells, HIF-1α cooperates with Sp1 to elevate expression of CD39 (Hart et al., 2010), which is also regulated by HIF-1β/ARNT and AhR receptor responses (Mascanfroni et al., 2015).

Hypoxia-dependent adenosine signaling also promotes angiogenesis, which has implications for invasion and metastasis. In human endothelial cells, hypoxic conditions enable HIF-2α to directly regulate adenosine A_2A_R, and HIF-1α to regulate A_2B_R, ENTs, and associated receptors. This mechanism promotes autonomous adenosine signaling and increased tissue vascularization (Li et al., 2020). In addition, adenosine impacts barrier-protective functions and RhoA activation in endothelial cells (Hassanian et al., 2014). Hence, in pancreatobiliary tumors, the adenosine rich stromal environment generated by elevated CD39 and CD73 enables neoplastic cells to escape CD8^+^ T-cell and NK cell immune surveillance (Ohta et al., 2006) and may facilitate cell invasion and metastatic dissemination by transiently increasing tumor vascularity (Ohta et al., 2006; Sun et al., 2010; Chiu et al., 2017; Kjaergaard et al., 2018).

Controversial roles of tumor-derived extracellular adenosine are emphasized at the interface of inflammation and cancer where transient or chronic hypoxic events play key roles in both inflamed areas of normal tissue and solid tumors. In this regard, the A_2A_R has been shown to protect normal tissues by promoting termination of inflammation but also support tumor promotion by protecting cancerous tissues from anti-tumor T cells. Tissue-protective mechanisms involve the hypoxia-driven accumulation of adenosine which, *via* cAMP, enhances A_2A_R expression downregulating the inflammatory response and preventing exacerbated tissue damage after injury. An important characteristic of this beneficial hypoxia-adenosinergic downregulation of activated immune cells is that it acts in a delayed-negative feedback manner, which is crucial for avoiding damaging ischemic events and for the protection of normal tissues from overactive immune cells (Ohta and Sitkovsky, 2001; Sitkovsky, 2009). Conversely, deleterious effects of A_2A_R signaling have been described that rely on diminished TCR signaling and IFN-gamma production (Ohta et al., 2006). Both events are triggered by hypoxic-driven elevated intracellular levels of cAMP which ends up misguiding anti-tumor T cells (Ohta et al., 2006).

Hypoxic conditions haves remarkable influences in the purinergic system by regulating CD39, CD73, and both the P1 and P2 receptors; thus, it is important to highlight potential therapeutic implications of weakening the hypoxia-A2-adenosinergic immunosuppressive pathway in the TME (Hatfield and Sitkovsky, 2020). Studies have shown that modulating hypoxic conditions improves anti-tumor effects in a metastatic model of orthotopically grown breast tumors (Hatfield et al., 2015). Moreover, increased oxygenation decreases levels of tumor-protecting extracellular adenosine and reduces expression of HIF-1α dependent tumor-protecting proteins (Hatfield et al., 2014).

## Inflammatory Bile Duct Diseases

Primary Sclerosing Cholangitis (PSC) is a cholestatic form of liver disease, characterized by inflammation, thickening, and abnormal fibrosis of the intrahepatic and extrahepatic bile ducts. Risk factors for PSC include gut dysbiosis and inflammatory bowel disease. PSC has the potential to evolve into biliary cirrhosis and is a preneoplastic condition, predisposing to cholangiocarcinoma, and colorectal cancer (Razumilava et al., 2011).

**Figure 2:**
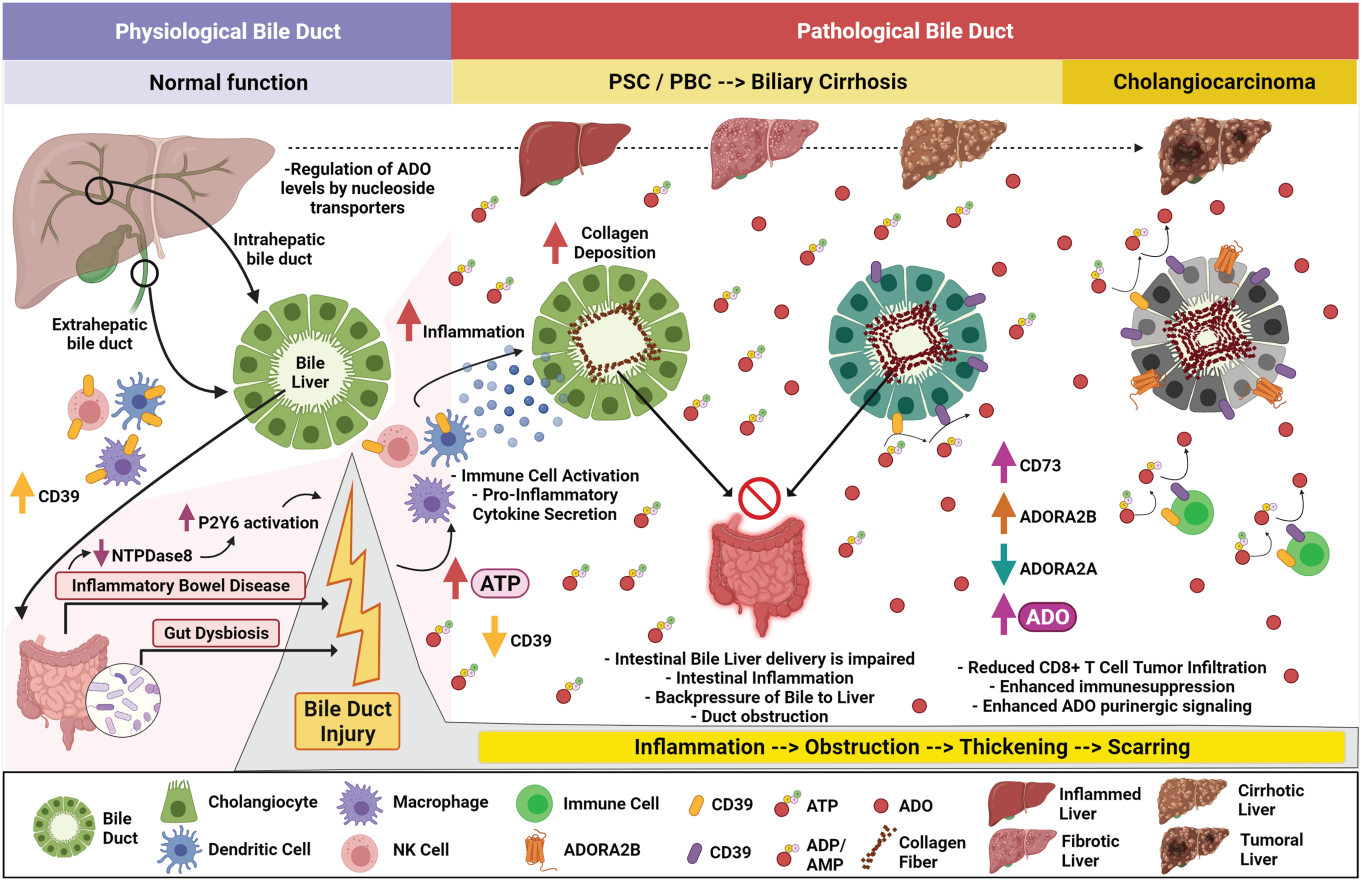
Purinergic signaling in cholestatic liver disease, biliary cirrhosis, and development of cholangiocarcinoma. Ectonucleotidase-expressing immune cells, pancreatic acinar cells, and cholangiocytes help balance gut-derived ATP levels arising within the hepatopancreatobiliary tract. During Inflammatory Bowel Disease (IBD) with associated gut dysbiosis, increased levels of ATP are secreted impacting physiological bile duct function and provoking further cholestasis. Decreased intestinal ENTPDase8 promotes enhanced P2Y_6_ activation and exacerbates the inflammatory phenotype. Immune cells may lose CD39 expression, allowing systemic ATP accumulation with activation of dendritic cells, macrophages, and NK cells. This effect is pronounced when in presence of CD39-deprived macrophages. The activated immune cells secrete pro-inflammatory signals when these cells arrive within the pancreatobiliary tract promoting cholangiocyte inflammation. Primary Sclerosing Cholangitis (PSC) and Primary Biliary Cholangitis (PBC) are associated with chronic ductular injury, impairing intestinal bile liver delivery, promoting backpressure to the liver, and exacerbating intestinal inflammation. Chronic inflammation and intra bile duct fibrosis trigger duct obstruction, thickening, and scarring which leads to biliary cirrhosis. Decreased A_2A_R and elevated CD73 and A_2B_R expression amplify adenosine signaling which reduces CD8^+^ T-cell tumor infiltration and enhances immune suppression, priming Cholangiocarcinoma initiation and development.

The role of CD39 in PSC was recently highlighted in a murine model of biliary injury and sclerosing cholangitis induced by multidrug resistance protein 2 (Mdr2) deficiency (Peng et al., 2017). Here, genetic deletion of CD39 resulted in higher levels of hepatic CD8^+^ T cells, liver injury, ductular reaction, and scarring. Loss of CD39 resulted in elevated ATP release in the gut, which activated DC and CD8^+^ T cells. Activated DC and CD8^+^ T cells then trafficked to the liver to target biliary epithelia, resulting in cholangitis and periductular fibrosis (Figure 2; Peng et al., 2017). These data are consistent with the complex mechanisms linking intestinal inflammation and PSC. Notably, the role of CD39 in limiting inflammation in this model is complicated by discordant impacts on ATP- and adenosine-mediated effects. As an example, subsets of bacterial species in dysbiosis activate immune cells, which upregulate CD39 and traffic to the liver. This mechanism increases intrahepatic levels of adenosine, perhaps resulting in aberrant immunosuppression, predisposing to cancers in cholestatic liver disease (Longhi et al., 2017; Tripathi et al., 2018; Vuerich et al., 2020).

To address this in the myeloid lineage, the role of CD39 in liver fibrosis has been studied in myeloid-specific CD39-deficient mice. After exposure to 3,5-diethoxycarbonyl-1.4-dihydrocollidine (DDC), myeloid-specific *CD39^−/−^* mice manifested worse liver fibrosis compared to wild-type mice, indicating CD39 myeloid expression is protective in sclerosing cholangitis (Rothweiler et al., 2019). These data indicate at early stages of biliary fibrosis, CD39 may be important for scavenging nucleotides and decreasing extracellular ATP levels to protect from sustained or chronic inflammatory signaling, but the consequences of sustained elevated CD39 in mediating long-term aberrant adenosinergic effects are to be still determined.

Primary Biliary Cholangitis (PBC) is an autoimmune cholestatic liver disease, which is characterized by damage to the intrahepatic bile ducts resulting in cirrhosis in some patients. PBC is slow progressing and associated with a number of malignancies including pancreatic, breast, and hepatocellular carcinoma. PBC is manifested by accumulation of bile in the liver, portal inflammation, and antimitochondrial antibodies (Ide et al., 2017). PBC can be managed with ursodeoxycholic acid (UDCA), a hydrophilic bile salt metabolized by the microbiome (Guarino et al., 2013). The proportions of CD4^+^CD39^+^ and CD8^+^ T regulatory cells, important for preventing autoimmune disease, are decreased in these patients (Lan et al., 2006; Bernuzzi et al., 2010) and CD4 + CD39+ cells are significantly reduced in the PBMCs of these patients compared to healthy controls. A mouse model for PBC has been described using 16-week chronic exposure to polyinosinic–polycytidylic acid (poly I:C). In these mice, similar to observed findings in human patients, CD39^+^CD4^+^ and CD39^+^ T regulatory cells are decreased in spleens and livers and CD39^+^ cells were decreased in PBMC. However, CD73^+^ cell numbers were not altered. In addition to loss of CD39, the A_2A_R, but not any other adenosine receptor, was decreased in the livers of poly I:C treated mice, indicating loss of hepatic A_2A_R and reduced anti-inflammatory adenosine signaling may be associated with early progression of PBC (Figure 2; Gong et al., 2021).

## Bile Duct Cancer

Tumors arising in the bile ducts are considered rare, yet highly lethal. Cholangiocarcinoma is considered aggressive and highly invasive with poor prognosis. This malignancy arises in the biliary tract and consists of two major subtypes: intra- vs. extrahepatic cholangiocarcinoma. HIF-1α levels are significantly elevated in cholangiocarcinoma cell lines compared to normal biliary cells and HIF-1α is important for cholangiocarcinoma cell line proliferation, migration, and invasion (Yu et al., 2020). ATP and adenosine have been shown to have anti-proliferative and anti-motility effects (Lertsuwan and Ruchirawat, 2017) in experiments conducted using cholangiocarcinoma cell (CCA) lines. Purinergic receptor expression levels in CCA cell lines were analyzed using qPCR and P2 receptors were expressed in CCA cell lines and in immortalized cholangiocytes, but in this study, adenosine receptors were not identified. Further elucidation of these mechanisms is needed, especially in the context of hypoxia, to evaluate the mechanistic consequences of elevated purinergic receptors in cholangiocarcinoma.

In contrast to *in vitro* studies, correlative immunohistochemical analysis of patients with cholangiocarcinoma in two separate cohorts has shown CD73 is elevated in cholangiocarcinoma and has prognostic implications. Immunohistochemistry in the first cohort showed CD73 staining in the majority of intra- and extrahepatic cholangiocarcinoma tissues. Notably, in normal hepatobiliary tissue, CD73 was expressed in the apical region of cholangiocytes and pancreatic ducts, and in the canalicular of hepatocytes (Sciarra et al., 2019). In the second cohort of 140 patients, elevated CD73 was highly correlated to lymphatic metastasis, tumor size and negatively associated with tumor-infiltrating CD8^+^ T cells. These data suggest that inhibition of CD73 is a promising modality for immunotherapy in patients diagnosed with intra- or extrahepatic cholangiocarcinoma (Xu et al., 2021).

In homeostatic biliary epithelia, the expression of hCNT3 is well represented and appears to be the major player in the extracellular regulation of adenosine levels. Moreover, as this receptor is regulated by A_2A_R, it contributes to complete purinergic control of bile flow which was started by ATP secretion into the bile (Godoy et al., 2014). On the other hand, in a subgroup of patients with biliary tract cancer, hENT1 expression correlated with overall survival, suggesting its participation in the intracellular transport of gemcitabine may play a role in predicting the subpopulation of patients who could benefit from this therapy (Santini et al., 2011).

## Inflammatory Pancreatic Diseases

Under normal physiologic conditions, pancreatic acinar cells express P2X1, P2X4, P2Y_2_, and P2Y_4_. The role of these P2R is thought to be related to signaling responses driven by secretion of ATP at the luminal side. In addition, intercalated pancreatic ducts expressing functional P2Rs, such as P2Y_2_, P2Y_4_, P2Y_6_, P2Y_11_, P2X4, and P2X7, respond and mediate ductal secretion of bicarbonate-rich fluid (Hayashi et al., 2016; Sciarra et al., 2019). ATP released by acini is hydrolyzed to ADP/AMP and adenosine by ectonucleotidase expression on ducts and both ADP and adenosine regulate ion channels on pancreatic ducts (Burnstock and Novak, 2012; Novak et al., 2020).

**Figure 3:**
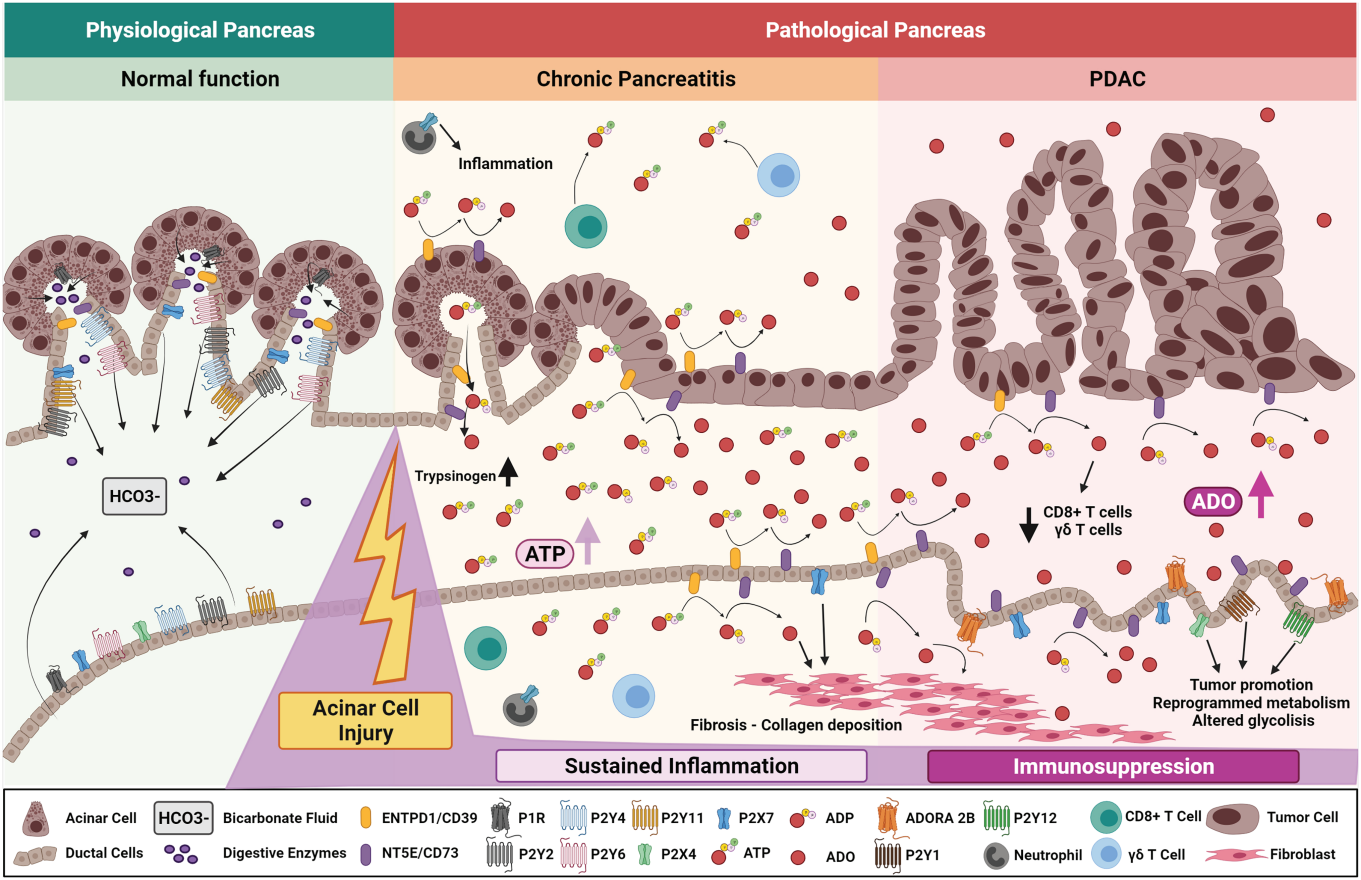
Purinergic signaling mechanisms in chronic pancreatitis and development of pancreatic cancer. Purinergic signaling mediates normal pancreatic function by regulating ductal P2Rs-dependent bicarbonate fluid release and acinar CFTR-dependent exocrine pancreatic secretion. After acinar cell injury, increased levels of ATP are secreted into the extracellular compartment primarily from acinar cells, increasing pancreatic trypsinogen levels and affecting normal pancreatic function. In time, sustained ATP-derived inflammation and adenosine generation stabilizes pancreatic chronic injury and/or ongoing pancreatitis. Increased expression of ductal CD73 elevates adenosinergic signaling triggering collagen deposition and further fibrotic development. Altered ductal cells undergo aberrant purinergic signaling though P2Rs affecting glycolytic metabolism and cumulative pancreatic ADO signaling promotes CD8^+^ T cell and ϒδ T-cell depletion. These factors promote an immunosuppressive environment ultimately priming the organ to malignant transformation, PDAC initiation, and progression.

In acute pancreatitis, injured acinar cells undergo autophagy or uncontrolled cellular death promoting the release of trypsinogen, cytokines, and ATP to the extracellular space allowing the activation and infiltration of several immune cells to the injured site (Sendler et al., 2018; Mayerle et al., 2019; Saluja et al., 2019). Heightened trypsinogen and ATP release elevates induction of acinar cell p38 MAPK and NF-κB pathways, which further contribute to pancreatic inflammation (Dixit et al., 2019). Elevated purinergic or adenosine signaling in pancreatic ducts results in dysregulation of bicarbonate secretion which alters the pH of pancreatic secretions provoking pancreatitis (Figure 3). In addition, purinergic receptors expressed on infiltrating neutrophils exacerbate pancreatitis. In a mouse model of acute pancreatitis, P2X1 expressed on neutrophils contributes to the inflammatory response and severity of pancreatitis (Wang et al., 2020). Expression of purinergic receptors and ectonucleotidases play an important role in mediating the physiological and pathological function of the pancreas, given their broad expression in epithelial, immune, and stromal cells (Novak et al., 2020).

Chronic pancreatitis is an important risk factor for development of pancreatic ductal adenocarcinoma and is characterized by acinar injury, damage of the gland, sustained inflammation, fibrosis, and loss of islet cells. Chronic pancreatitis manifests in unrelenting abdominal pain, malnutrition, pancreatogenic diabetes (type 3c diabetes), and exocrine and endocrine insufficiency (Bhattamisra et al., 2019; Singh et al., 2019; Illés et al., 2020; Wei et al., 2020; Cruz-Monserrate et al., 2021). Risk factors include heavy alcohol abuse, tobacco smoking, and genetic predispositions (Majumder and Chari, 2016; Singh et al., 2019). Events that promote sustained pancreatic injury, like chronic pancreatitis, result in excessive accumulation of ATP which dysregulates the physiological state of the gland and may be a key driver of neoplasia.

*In vitro* cultures of ethanol-induced toxicity on pancreatic ductal epithelial cells exposed to micromolar concentrations of ATP/ADP resulted in a protective effect *via* the P2Y_1_ receptor. ATP/ADP activation of the P2Y_1_ receptor increased intracellular levels of cAMP which is an important mechanism for maintenance of ductal epithelial integrity in the presence of ethanol (Seo et al., 2016). While a protective mechanism through P2R purinergic signaling has been studied in pancreatic ducts, the role of adenosine signaling in alcohol-associated pancreatitis has not been described.

In addition, the role of purinergic signaling in the development of pancreatogenic or secondary type 3C diabetes has not been established (Hart et al., 2016). Experiments evaluating the function of islets in models of insulin resistance, diabetes, and obesity have shown increased production of ATP in diabetic and obese mice. This has been shown to reduce anti-inflammatory secretion of IL-10 from islet-associated macrophages indicating increased ATP may have a pathogenic role in development of type 3C diabetes (Weitz et al., 2020). Adenosine signaling also regulates ß-cell survival and regeneration in inflammatory microenvironments as well as regulates insulin secretion and lipid homeostasis through P1 receptors (Antonioli et al., 2015).

Global deletion of CD39L (*Entpd3^−/−^*) and islet-specific deletion of CD39L using *Entpd3^flox/flox;InsCre^* mice have been studied as this ENTPD is the dominant islet and β cell ectonucleotidase. *Entpd3^−/−^* mice are resistant to high fat diet-induced obesity and have elevated basal metabolic rates, when fed a high fat diet associated with improved glucose tolerance. This phenotype was associated with higher uncoupling protein 1 (UCP-1) in brown adipose tissue. Studies on *Entpd3^flox/flox;InsCre^* mice show a similar phenotype indicating Entpd3 in β cells is not protective against diet-induced obesity and insulin resistance (Sandhu et al., 2021). The potential role of purinergic and adenosine signaling in pancreatogenic diabetes has a number of therapeutic implications and should be further explored.

## Ductal Adenocarcinoma of the Pancreas

Pancreatic ductal adenocarcinoma (PDAC) is one of the world’s most lethal malignancies. Risk factors include age, smoking, inherited predisposition due to a germline mutation, obesity, long-standing diabetes, chronic pancreatitis, and type 3 C diabetes (Xiao et al., 2016; Saluja and Maitra, 2019; Hart and Conwell, 2020).

Several purinergic P2Rs are widely expressed in pancreatic cancer cell lines (Hansen et al., 2008). Among these, P2X5, was found elevated in human PDAC and associated with malignant behavior of cancer cells; however, the role of P2X5 in pancreatitis and tumor development is not yet well stablished (Zaccagnino et al., 2016) and there remains uncertainty regarding the role of P2X7 in pancreatitis and PDAC initiation.

An *in vivo* study using P2X7 inhibitors revealed a tumor-promoting function for this receptor and highlighted its participation in stellate cell fibrosis and collagen deposition (Giannuzzo et al., 2016). Other P2 receptors including P2Y_1_, P2Y_6_, and P2Y_12_ were identified as increased in PDAC cell lines and exerted tumor-promoting functions in the presence of ATP (Qadir et al., 2018; Novak et al., 2020). Studies show that P2Y_2_ is associated with poor prognosis and its activation promotes PDAC cancer progression by reprogramming cancer cell metabolism and glycolysis (Hu et al., 2019). P2Y_2_ effects were prevented when genetically or pharmacologically inhibited, providing enhanced evidence regarding mechanisms of ATP-mediated PDAC progression through P2Y_2_ receptor on malignant epithelium (Choi et al., 2013; Hu et al., 2019).

Adenosine signaling is emerging as a critical mediator of PDAC. Evaluation of adenosine P1 receptors from gene expression analysis of PDAC tissues based on The Cancer Genome Atlas (TCGA) show that both *ADORA2A* and *ADORA2B* expression are increased in PDAC compared to normal tissues. Of interest, *ADORA2B* is a marker of poor prognosis and *ADORA2A* has better overall survival prognostic impact (Hayashi, 2019). Another well-studied receptor in this disease is A_3_R, which promotes the tumorigenic phenotype *via* ERK1/2 signaling pathway (Gorzalczany et al., 2017).

In addition to P1 receptors, CD73 is elevated in pancreatic cancer (Chen et al., 2020; Zhao et al., 2021). In pancreatic cancer, adenosine generation mediated by the concerted activity of CD39 and CD73 is correlated with lower infiltration of CD8^+^ T cells and γδ^+^ T cells, as well as stellate cell proliferation and collagen production (Künzli et al., 2008; Chen et al., 2020; Shevchenko et al., 2020). While correlative, these data indicate that adenosine signaling promotes pancreas fibrotic development by modulating ECM remodeling and immunosuppression (Blocking CD73 Can Shrink Pancreatic Tumors, 2021).

High expression of CD73 has been described in tumor cells in two independent cohorts of PDAC patients (Chen et al., 2020; Zhao et al., 2021). Elevated CD73 is associated with a decrease in CD4^+^, CD8^+^, and CD21^+^ tumor-infiltrating lymphocytes and is associated with aggressive clinical behavior (Zhao et al., 2021). Histologic evaluation of CD73 in pancreatic and PDAC tissues has shown CD73 is expressed in pancreatic ducts and in PDAC tissue, but not acinar cell carcinoma (Sciarra et al., 2019). In genetically engineered mouse models of PDAC, CD73 is highly expressed on neoplastic and invasive lesions arising in pancreatic ducts and high CD73 is associated with high adenosine levels in pancreata from these mice. Inhibition of adenosine generation decreased tumor growth in spontaneous and established models and is correlated with increased activated CD8^+^, CD4^+^ T cells and macrophages, implicating anti-CD73 immunotherapy in PDAC patients with high CD73 expression (Singh et al., 2021). Further studies are needed to fully comprehend the molecular mechanisms underlying CD73 function and adenosine generation in pancreatitis and PDAC microenvironment modulation. CD39 and CD39L transcripts are both increased in chronic pancreatitis and pancreatic cancer. Pathologic analysis reveals both are localized in vascular and stromal elements. In contrast to CD73, high expression of CD39 is significantly associated with better long-term survival in PDAC (Künzli et al., 2007).

Nucleoside transporter expression was found altered during the progression from normal pancreatic epithelia to a malignant state. Evidence suggests that functional hENTs may result in increased gemcitabine uptake by pancreatic cells. Hence, reduced expression of hENT1 implicates a link between protein expression levels and chemoresistance (Nakano et al., 2007; Carter et al., 2021). Moreover, in PDAC patients, its low expression correlates with a significant reduction in progression-free survival and disease-free survival when compared to patients with medium to high levels of hENT1 expression (Farrell et al., 2009). Aberrant expression of hENT2 was also observed in pancreatic cancer cells and suggested to contribute to chemoresistance; however, its participation is not yet fully elucidated (Alvarellos et al., 2014). On the contrary, hCNT3 expression in pancreatic tumors correlates with overall patient survival, with an increased expression of the transporter usually associated with a longer overall patient survival. The aberrant expression of hCNT3 was observed in pancreatic tumors and pancreatic cancer cell lines and is of high relevance for pancreatic cancer patients given its ability to transport a large variety of nucleoside-derived drugs and, more importantly, gemcitabine for solid tumors (Stecula et al., 2017).

## Therapeutic Considerations in Pancreatobiliary Cancers

Therapeutic intervention has been proposed to correct the pathophysiological levels of extracellular nucleotides and nucleosides to restore CD39 or P2 receptor functionality in chronic inflammatory states. Given space constraints and the important recent developments in purinergic immunotherapy and check point inhibition in cancer, may we refer the interested reader to several reviews that cover the topics of augmenting adenosinergic pathways and blocking ATP-mediated pathways in the liver, pancreas, and GI tract in several inflammatory disease states in order to limit cancer development (Eltzschig et al., 2012; Burnstock et al., 2014; Longhi et al., 2017, 2021; Vuerich et al., 2020).

Given the complex expression of purinergic mediators in epithelial, stromal, and immune compartments, receptor blockade may not only impair the specific signaling pathway but also modulate the tumor microenvironment impacting other purinergic receptors. Preclinical studies targeting the purinergic system suggest that combined inhibition of more than one member of the pathway may enhance anti-tumor immune responses (Young et al., 2016; Moesta et al., 2020).

Firstly, metastatic dissemination and colonization are complex processes linked to spread of tumor cells. *In vivo* lineage tracing experiments indicate circulating pancreatic neoplastic epithelial cells disseminate prior to establishment of a primary tumor. In these *in vivo* experiments, inflammation appears as a potent driver of epithelial dissemination or delamination (Rhim et al., 2012, 2014; Hendley et al., 2016). Defining the role of purinergic signaling coordinated between neoplastic epithelial cells, innate or adaptive immune cells and the foreign parenchymal microenvironment will be crucial to determining whether immune mechanisms prevail and eliminate tumor cells before these cells spread.

Secondly, ectonucleotidases, P2 and P1 receptors are predicted to play central roles in shaping the foreign microenvironment by boosting ATP/AMP/adenosine conversion, favoring an immunosuppressive niche. P2X1 negative neutrophils are key players in the invasion of foreign liver tissues. After being mobilized and recruited to metastatic sites, P2X1 negative neutrophils exert immunosuppressive activities *via* Nrf2-supported mitochondrial metabolism and predispose the foreign tissue as a suitable site to successful metastatic colonization (Wang et al., 2021b). In addition, purinergic signaling impacts T-cell memory and exhaustion. These prior findings indicate that targeting CD39 has merit for augmenting checkpoint therapies for treating cancer and chronic infections (Sun et al., 2010; Deaglio and Robson, 2011). These observations suggest that specific mechanisms operate to modulate purinergic responses in memory T cells. The response of memory T cells to adenosine and specific receptor agonists might be modulated at the level of intracellular cyclic-AMP and the signaling pathways it controls (Peter et al., 2007). Furthermore, given the recent discovery of CD39^+^CD8^+^ T cells in regulating metastatic dormancy, understanding the mechanisms of purinergic signaling in metastasis is critical especially given the predicted duration of time from primary tumor to metastatic disease in pancreatic cancer (Yachida et al., 2010). These experiments also infer major implications for CD39 inhibitors, which may block metastatic spread of cancer cells in experimental models (Sun et al., 2010).

In the context of checkpoint inhibitors, targeting CD39 is likely to be more effective than single agents targeting CD73 (Beavis et al., 2012), due to reduced generation of immunosuppressive adenosine, and also promoting the accumulation of immunostimulatory ATP (Martins et al., 2009; Vieira et al., 2014). ATP in the tumor microenvironment amplifies TCR signaling in lymphocytes triggering anti-tumor CD8^+^ infiltration and activation (Boison and Yegutkin, 2019). In addition, sustained ATP promotes long-term memory CD8^+^ T cells critical for adaptive immunity. CD39 blockade has been shown to reduce tumor growth not only because of the diminished generation of downstream adenosine (see below) but also by enhanced P2X7-mediated NRLP3 inflammasome due to extracellular accumulation of ATP (Li et al., 2019; Baeza-Raja et al., 2020). In models of melanoma metastasis, tumor growth in the liver is substantively inhibited in mice with CD39 null vasculature or CD39 null bone marrow-derived cells. CD4^+^FoxP3^+^ Tregs expressing CD39 repressed anti-tumor immunity by NK cells and pharmacologic inhibition of CD39 significantly limited melanoma tumor growth. Thus, CD39 expression on Tregs promotes metastatic growth and targeting this may provide a strategy for secondary hepatic malignancies (Sun et al., 2010).

In contrast, adenosine is generated by CD39-CD73 expression on Treg and memory T cells and inhibits effector T-cell immunity, which opposes effects of ATP. Adenosine receptor stimulation (A_2A_R or A_2B_R) on macrophages restrains production of nitric oxide and pro-inflammatory cytokines. Thus, adenosine receptors resolve inflammation and promote tissue repair, yet in chronic inflammation and neoplasia, cumulative adenosine signaling promotes transformation and immunosuppression (Boison and Yegutkin, 2019).

When targeting adenosine receptors, it is important to consider that A_1_R and A_2_R have the capacity to form heteromers, which implies a cross-communication between Gi and Gs proteins (Navarro et al., 2018). These structures were shown to act as sensors of adenosine concentration and modulate adenosine signaling in such a way that there is either A_1_R or A_2_R-mediated effects. Similar properties were described for heteromers formed between A_2A_R and A_2B_R (Franco et al., 2021). Thus, these recent findings highlight the need to elucidate whether heteromer formation is found in pancreatobiliary cancers, as studying its peculiar signaling dynamic will be essential to understand adenosine-mediated effects and potentially propose them as targetable structures.

A challenging aspect when targeting purinergic or adenosinergic receptors is most physiological and preclinical studies to unravel receptor function and therapeutic effects have been carried out in rodents, which, do not necessarily represent the affinity and functioning of human receptors. Hence, several compounds in preclinical platforms were selected based on their affinity to human receptors, which do not necessarily represent similar rodent receptor interaction. Indeed, this aspect was analyzed for adenosine receptors and determined that for some ligands the potency and selectivity are species-dependent and proposed that a comprehensive characterization of compounds and species-specific affinities are key to understand whether they may be suitable ligands to pursue drug therapy in the clinic (Alnouri et al., 2015).

Beneficial effects were observed when targeting CD73 and A_2A_R alone or in combination with PD-1/PD-L1 inhibitors (Allard et al., 2013; Mittal et al., 2014; Beavis et al., 2015; Hay et al., 2016). Moreover, preliminary results of the first phase 1/2a study of a CD73 inhibitor in combination with the PD-1 inhibitor nivolumab induced partial responses or stable disease in 28% of patients with various malignancies (NCT02754141). Another phase 1 clinical trial recently evaluated simultaneously inhibiting CD73 and PD-L1 in subjects with select advanced solid tumors (NCT02503774), however, results remain to be published.

Currently, several phase I/II studies are evaluating CD39 (NCT04336098) and A_2A_R blockade in combination with PD-1 (NCT03884556, NCT03207867), PD-L1 (NCT02655822) inhibitors, or with standard chemo- or immunotherapy (NCT04306900). Besides, in locally advanced or recurrent/metastatic PDAC, several phase I clinical trials are being conducted by targeting CD73 alone (NCT04148937) or in combination with PD-1 (NCT04104672) and A_2A_AR inhibitors (NCT03549000, NCT03454451; Table 1).

**Table 1:** Clinical trials evaluating the potential use of purinergic mediators as targets for hepatopancreatobiliary tumors.

Target	Drug +/− combination therapy or Outcome	Tumor	Identifier	Study phase	Sponsor company
A2AAR	NIR178 (A2AAR antagonist) in combination with PDR001 (anti-PD-1 Ab)	Patients with solid tumors (including pancreatic cancer) and Non-Hodgkin Lymphoma	NCT03207867	Phase II	Novartis Pharmaceuticals
A2AAR	Ciforadenant (A2AAR inhibitor) in combination with Atezolizumab (PD-L1 inhibitor)	Patients with selected incurable tumors	NCT02655822	Phase I/Ib	Corvus Pharmaceuticals
CD39	TTX-030 (anti-CD39 Ab) in combination with standard chemo- or immunotherapy	Patients with advanced tumors	NCT04306900	Phase I/Ib	Trishula Therapeutics
CD39	SRF617(anti-CD39 Ab)	Patients with advanced solid tumors	NCT04336098	Phase I	Surface Oncology
CD39	TTX-030 (anti-CD39 Ab) +/− anti-PD-1 immunotherapy	Patients with Lymphoma or solid tumors	NCT03884556	Phase I	Trishula Therapeutics
CD39	Monotherapy	Patients with advanced solid tumors	NCT05234853	Phase I	Purinomia Biotech, Inc
CD73	LY3475070 (CD73 inhibitor) +/− Pembrolizumab	Patients with advanced solid tumors including PDAC	NCT04148937	Phase I	Eli Lilly and Company
CD73 +/− A2AAR	NZV930 (anti-CD73 Ab) +/− PDR001 (anti-PD-1 Ab) +/− NIR178 (A2A antagonist)	Patients with advanced solid tumors including PDAC	NCT03549000	Phase I/Ib	Novartis Pharmaceuticals
CD73 +/− A2AAR	CPI-006 (anti-CD73 Ab) +/− ciforadenant (oral A2A inhibitor) +/− pembrolizumab (anti-PD1 Ab)	Patients with selected advanced solid tumors including PDAC	NCT03454451	Phase I/Ib	Corvus Pharmaceuticals
CD73 +/− PD-1	AB680 in combination with Zimberelimab (AB122), nab-paclitaxel and gemcitabine	Patients with advanced PDAC	NCT04104672	Phase I	Arcus Biosciences

Lastly, the role of microbiome in regulating systemic ATP or adenosine signaling in metastasis warrants further analysis. Inosine derived from the microbiome was recently described as a key mediator of immunosuppression and response to checkpoint inhibitor immunotherapy (Mager et al., 2020) and analysis of human PDAC microbiome has revealed certain bacterial species are correlated with overall survival (Riquelme et al., 2019). However, the role of gut bacteria in altering local and systemic purinergic and adenosine signaling in pancreatic diseases has not been well defined.

## Conclusion

Purinergic signaling pathways have important roles in initiation, progression and resolution of inflammation, yet sustained activation appears to disrupt normal tissue homeostasis and promote chronic inflammatory conditions and scar formation. These factors elevate the risk of end-organ failure and development of pancreatobiliary cancer. Beneficial ATP inflammatory P2R-mediated effects might be overshadowed in chronically inflamed tissues or neoplastic environments, where enzymatic conversion of nucleotides through elevated CD39 and CD73 may lead to excessive adenosine accumulation. Instead of promoting resolution of inflammation and tissue repair, this rather promotes A_2B_R-mediated or other aberrant pathways important for cellular transformation and immune suppression. In this context, it should be kept in mind that outcomes are not only determined by extracellular bioavailability of ATP and its nucleoside derivatives, but also by the expression of other purinergic mediators and P1 or P2 receptor functionality.

Therapeutic strategies targeting CD39 or CD73 as well as A_2B_R antagonism have the potential to reverse adenosine-mediated immunosuppression, albeit only CD39 blockade will boost ATP-mediated anti-tumor immunity. However, the widespread expression of CD39, CD73, and purinergic receptors on immune cells, myofibroblasts, and epithelial cells complicates understanding of the mechanistic basis for purinergic and adenosine signaling during chronic inflammation, fibrosis, and tumor formation.

We propose that modulation of purinergic signaling represents novel avenues for reversing inflammation, and by virtue of limiting scar formation and cell transformation, decreasing the risk of cancer. Once cancer has developed, then immunotherapy strategies for treatment of pancreatobiliary malignancies show great promise. Dissecting out the implications of these propositions and determining the clinical timing of these divergent approaches will require further study, attention to personalized medicine, and innovative clinical trials.

## Author Contributions

EF and JMB-L wrote the main manuscript file. CJ, SCR, and HKE edited the manuscript file. All authors contributed to the article and approved the submitted version.

## Funding

NIH R21CA249924 and DK056338-18 (Pilot) to JMB-L. NIH grants R01HL154720, R01DK122796, R01HL133900 and Department of Defense W18XWH2110032 to HKE. NIH R01DK122708, R01DK121330 and R01DK122796 to CJ. NIH R01DK108894; R21CA221702, Emerson Collective Cancer Research Fund Award and Department of Defense Award W81XWH-16-0464 to SCR.

## Conflict of Interest

SCR is a scientific founder of Purinomia Biotech Inc and has consulted for eGenesis. AbbVie and SynLogic Inc; his interests are reviewed and managed by HMFP and Beth Israel Deaconess Medical Center in accordance with the conflict-of-interest policies.

The remaining authors declare that the research was conducted in the absence of any commercial or financial relationships that could be construed as a potential conflict of interest.

## Publisher’s Note

All claims expressed in this article are solely those of the authors and do not necessarily represent those of their affiliated organizations, or those of the publisher, the editors and the reviewers. Any product that may be evaluated in this article, or claim that may be made by its manufacturer, is not guaranteed or endorsed by the publisher.
